# Using texture analysis of ultrasound images to assess the effect of cupping therapy on muscle quality of the triceps

**DOI:** 10.1371/journal.pone.0301221

**Published:** 2024-03-27

**Authors:** Isabella Yu-Ju Hung, Yih-Kuen Jan

**Affiliations:** 1 Department of Nursing, Chung Hwa University of Medical Technology, Tainan, Taiwan; 2 Department of Kinesiology and Community Health, University of Illinois at Urbana-Champaign, Urbana, Illinois, United States of America; Università degli Studi di Milano: Universita degli Studi di Milano, ITALY

## Abstract

The objectives were to investigate whether cupping therapy improves muscle quality and the interaction between duration and negative pressure of cupping therapy affects muscle quality. A 2×2 factorial design with repeated measures was used to examine the efficacy of cupping therapy on improving muscle quality. The independent factors were cupping pressures at −225 and −300 mmHg and cupping durations at 5 and 10 min, and the dependent factor was texture of B-mode ultrasound image of the triceps. Four cupping protocols were applied to 12 participants at 4 different days. Texture analysis including contrast, correlation, energy, and homogeneity was applied to assess muscle quality from 480 ultrasound images. The two-way repeated measures ANOVA showed that there was an interaction between the pressure and duration factors on the superficial layer of the triceps in contrast (F = 5.434, *P* = 0.004) and correlation (F = 6.274, *P* = 0.029). In contrast texture, the superficial layer of the triceps showed a significant increase in three protocols: −225 mmHg for 5 min (1.0434 ± 0.130), −300 mmHg for 5 min (1.0339 ±0.1407), and −300 mmHg for 10 min (1.0563 ±0.1432) except −225 mmHg for 10 min (0.9704 ±0.0985). In correlation texture, the superficial layer of the triceps showed a significant decrease in all protocols: −225 mmHg for 5 min (0.9556 ± 0.07), −225 mmHg for 10 min (0.9831 ± 0.0708), −300 mmHg for 5 min (0.9976 ± 0.055), and −300 mmHg for 10 min (0.9406 ± 0.0809). The results indicate that the interaction between the pressure and duration factors of cupping therapy significantly increases contrast texture and significantly decreases correlation texture of the superficial layer of the triceps after cupping therapy. Cupping therapy decreases homogeneity among soft tissues of the treated muscle.

## Introduction

Cupping therapy has been widely used to manage various musculoskeletal impairment, including non-specific low back pain, carpal tunnel syndrome, plantar fasciitis, fibromyalgia, osteoarthritis, and delayed onset muscle soreness [1–3]. However, an evidence-based review indicates that cupping therapy has only low to moderate evidence for treating musculoskeletal impairment [4]. Insufficient evidence for cupping therapy may be attributed to the lack of mechanisms of action and the dose-response relationship of cupping therapy, such as the cup size, treatment duration, and magnitude of negative pressure [5, 6]. In the literature, most studies do not report the details of cupping therapy intensity (eg. the magnitude of negative pressure and the cup size). For example, the use of a cup not large enough (eg. 35-mm in diameter) for cupping therapy may not reduce muscle stiffness (eg. the triceps) [7]. Insufficient control of cupping doses may contribute to conflicting outcomes of cupping therapy trials [7–9].

Various theories have been proposed to explain benefits associated with cupping therapy [5, 6]. For example, alleviation of pain associated with cupping therapy may be attributed to changes in biomechanical properties of the skin by the Pain Gate Theory, Diffuse Noxious Inhibitory Controls, and Reflex Zone Theory. Removal of regional toxins and wastes could be explained by the Blood Detoxification Theory. Muscle relaxation and increased blood circulation may be explained by the Nitric Oxide Theory. Although the exact mechanism of action of cupping therapy has not been established, it is generally accepted that several physiological mechanisms could be involved for improving health after cupping therapy [5, 6].

The increased interest from the general public on cupping therapy is because of the appearance of cupping marks from elite athletes, such as Michael Phelps and Russel Westbrook. Subsequently, more research studies have been conducted to explore various outcome measures to shed light on potential physiological mechanisms of cupping therapy in the past decade [5, 6]. These studies have been focusing on the microvascular response related to the Nitric Oxide Theory and Blood Detoxification Theory of cupping therapy, and only few studies investigated the soft tissue (ie. the skin, subcutaneous tissue and muscle) response to cupping therapy [7, 8, 10]. The study of structural and biomechanical property of the soft tissue in response to cupping therapy could be an essential part of understanding the effectiveness of cupping therapy [7, 11]. Recently, several studies have been exploring the biomechanical responses of muscles after cupping therapy, such as myofascial pain syndrome, muscle stiffness, and tenderness [7, 12]. Jan et al. demonstrated that cupping therapy was more effective in reducing stiffness on the deep layer of the treated soft tissue. The superficial layer of the soft tissue measured from elastographic ultrasound consists of the skin, subcutaneous tissue, fat and muscle, and the deep layer consists of mainly muscles. The skin, subcutaneous tissue and fat have a relatively uncompressible mechanical property compared to the deep layer mainly consisting of muscles [7]. The finding is consistent with previous studies that the deep layer of the triceps could significantly change stiffness under different situations [13–15]. Therefore, cupping therapy may cause a vasodilatory response in the cutaneous for increasing blood flow to the cells, reduce muscle stiffness, and change tension and perception (pressure pain threshold) surrounding nerves and injured tissues for reducing pain. Based on these findings, the investigation of the mechanical property of the muscle is important to better understand the effect of cupping therapy.

Diagnostic ultrasound, as a routine clinical tool, can be used to assess soft tissue health. By investigating structural arrangement, mechanical property (eg. stiffness), and detecting latent inflammation [16–20], it is reasonable to speculate that ultrasound could be used to shed light on benefits of cupping therapy on managing musculoskeletal impairment. The current limitation of ultrasound practice is related to variations in measurement because of different operations of ultrasound (eg. different probe angles and different ultrasound gain settings) [21]. Muscle echo intensity has been demonstrated to be affected by the setting of the ultrasound device [22]. Recent advances in medical imaging processing, for example, texture analysis, could overcome the limitation of using ultrasound to characterize muscle composition and quality [23]. Texture analysis involves the mathematical representation of pixel intensity and spatial distribution within the designated region of interest (ROI) [23]. These mathematical features can be utilized to describe the underlying texture structure of the soft tissue and its association with different structural manifestations, including tissue damage and remodeling. Structural variation in muscle tissue or changes in intramuscular fat content can be observed with a quantitative image texture analysis. Image texture is composed of repetitive elements called primitives. The method of texture analysis is to investigate these primitives and following that distinguish the texture type with texture features. Texture analysis of ultrasound images may be used to quantify the property of the muscle, such as roughness, homogeneity, and structural complexity of the specific ROI within soft tissues (eg. areas treated by cupping therapy) [21]. The application of texture analysis of medical images of the muscle has been demonstrated to be sensitive to characterize changes in muscle composition and quality in pathophysiological conditions and aging [23–25].

Martinez-Paya et al. (2018) used texture analysis of muscle ultrasound images to assess the progression of amyotrophic lateral sclerosis (ALS) [26]. The authors found reduced granularity in the muscle of people with ALS and indicated that texture analysis of muscle ultrasound images was a promising biomarker to estimate neuromuscular function in people ALS. Sikio et al. evaluated the muscle texture by gray level co-occurrence matrix (GLCM) from magnetic resonance images (MRI) of the thigh muscles from athletes of different training, and detected muscle structural differences on specific exercise-load demands [27]. The authors used four co-occurrence parameters for each ROI, including angular second moment, inverse difference moment, entropy and difference entropy [27]. The first two were used to detect the image homogeneity, whereas the last two parameters were used to measure of the image complexity. ROI were defined as squares of 15×15 pixels, which were placed on the median of muscle cross-section to avoid partial volume effect on ROIs caused by contamination with the connective tissue around the muscle and visible fascicles. In an exercise-induced muscle damage studies, Matta et al. adopted two GLCM texture parameters, contrast (CON) and correlation (COR), and echo intensity (EI) on ultrasound images, and found that the elbow flexors muscle structural changes after eccentric training [24]. These studies have demonstrated that texture analysis is sensitive in characterizing changes in muscle composition and quality that are related to muscular performance in various pathophysiological conditions. Although the exact physiological and clinical meaning associated with these features extracted by texture analysis has not been fully established, texture analysis of muscle ultrasound images has demonstrated its potential as a noninvasive tool for characterizing muscle quality in various neuromuscular and musculoskeletal impairment as well as therapeutic efficacy, including the characterization of the effect of cupping therapy on the changes of muscle quality.

Alterations in structure and mechanical property caused by interventions, such as cupping therapy, can lead to variations in the acoustic characteristics of treated soft tissues, resulting in discernible differences in the texture patterns that could be detected by ultrasound images with texture analysis. Previous studies have demonstrated that pathological conditions are associated with a decrease in homogeneity of the soft tissue including the skin, subcutaneous tissue and muscle and exercise training can lead to an improvement in homogeneity of the soft tissue [23–25]. Because cupping therapy is reported to reduce musculoskeletal impairment [4, 10, 28], it is reasonable to assume that cupping therapy could reduce heterogeneity and improve homogeneity of the soft tissue that can be quantified by the use of texture analysis of muscle ultrasound images. The increased homogeneous property may be partly associated with the benefits of cupping therapy. Therefore, the general hypothesis of this study was that cupping therapy could reduce heterogeneity and improve homogeneity of the treated muscle. We hypothesized that: 1) cupping therapy improves the homogeneous property of the muscle that can be quantified by texture analysis of muscle ultrasound images, and 2) the interaction between cupping duration and cupping pressure is more effective on improving the homogeneous property of the muscle than only the cupping duration or cupping pressure. The results of this study could provide initial evidence for cupping therapy on improving muscle quality quantified by texture analysis of B-mode ultrasound images. To the best of our knowledge, this is the first study using texture analysis of ultrasound images to characterize the effect of cupping therapy on the muscle.

## Methods

A 2×2 factorial design with repeated measures was carried out in this study. The participant was blinded to the cupping protocol to minimize potential influences from psychological factors. The independent variables were the cupping pressure and cupping duration, and their interaction effect (cupping pressure × cupping duration). The dependent variable is the texture of the B-mode ultrasound image of the treated area. The negative pressure factor included −225 and −300 mmHg, and the duration factor included 5 and 10 min. There were four protocols for cupping therapy including: (A) −225 mmHg for 5 min, (B) −225 mmHg for 10 min, (C) −300 mmHg for 5 min, and (D) −300 mmHg for 10 min. In order to counterpoise the order effect, we utilized the counterbalanced design. Each protocol was tested in different days separately by 2–4 days. The orders of 4 protocols used in this study included: S1 (A, B, C, D), S2 (B, A, C, D), S3 (A, B, D, C), S4 (B, A, D, C), S5 (C, D, A, B), S6 (D, C, A, B), S7 (C, D, B, A), S8 (D, C, B, A), S9 (A, D, B, C), S10 (B, C, A, D), S11 (A, D, C, B) and S12 (B, C, D, A).

This was part of a larger project investigating microvascular and biomechanical responses following cupping therapy [29]. The data collection was performed between December 3, 2019 and July 12, 2022. This human subject research was approved by the University of Illinois at Urbana-Champaign Institutional Review Board (#20334).

### Participants

The inclusion criteria of this study were healthy people without any diagnosed diseases and aged from 18 to 30 years. The exclusion criteria were as the following: non-blanchable response of the red skin areas among the triceps muscle area (dominant side), scar or tattoo over the tested area, open wounds, cardiovascular disease, and smoking history within 3 years. The volunteers were enrolled in the study through flyers and word of mouth methods from the University of Illinois. Every subject has signed the informed consent before participating in the experiment. Because no prior studies on the texture analysis of ultrasound images after cupping therapy, the sample size estimation was based on our previous study indicating that cupping therapy was effective on reducing muscle stiffness [29]. Thus, a very large effect size (0.4) [30] and the power of 0.8 were chosen for achieving a sample size of 12 for this study.

### Instrumentation

**Fig 1:**
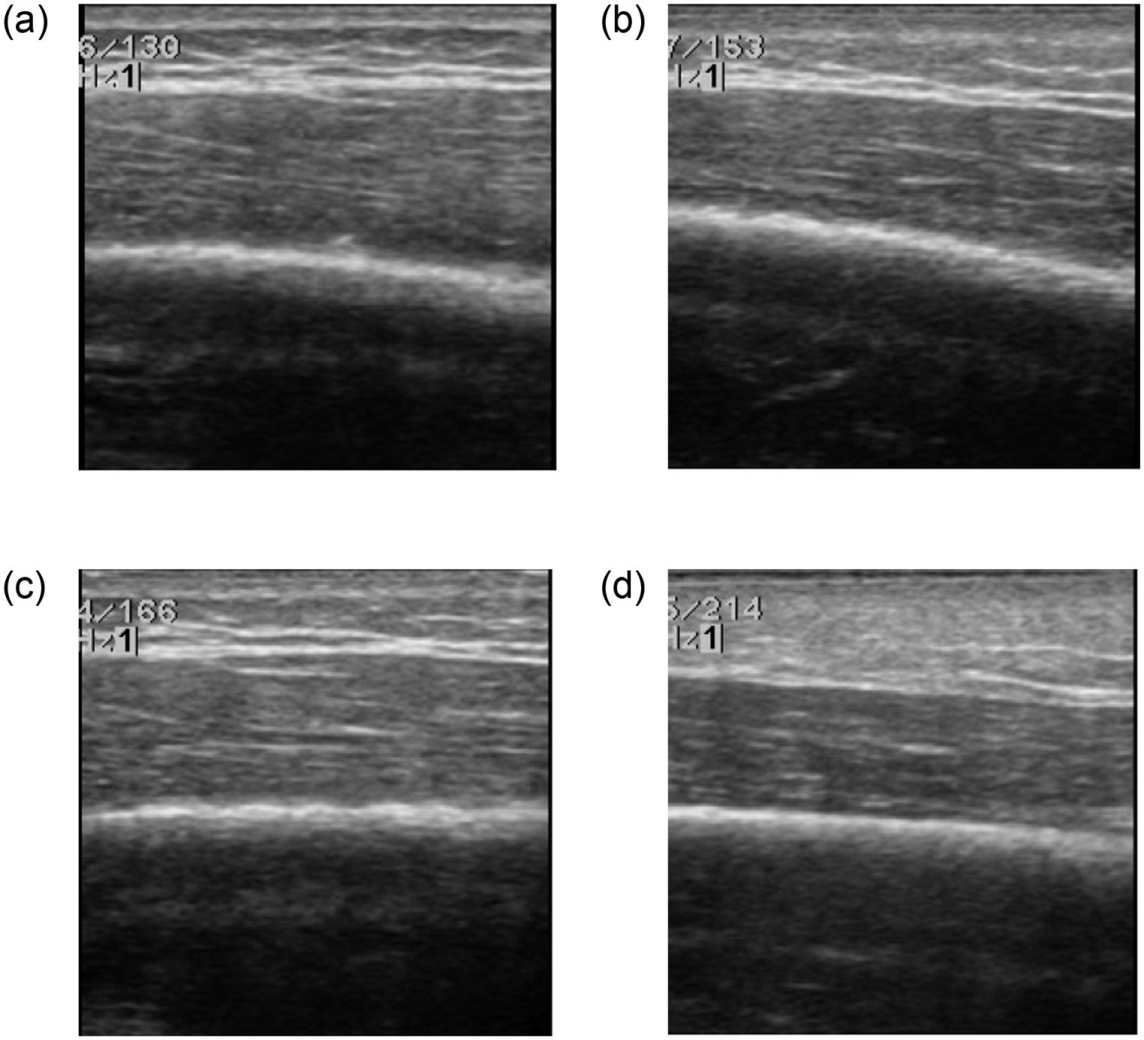
Typical examples of gray-scale ultrasound images before and after cupping therapy from a person. The changes between pre-cupping (a & c) and post-cupping (b & d) are not easily described by visual examinations. (a) Ultrasound image of the triceps of the dominant side before applying cupping therapy at −225 mmHg for 5 min. (b) Post-cupping ultrasound image from the condition of (a). (c) Ultrasound image of the triceps of the dominant side before applying cupping therapy at −225 mmHg for 10 min. (d) Post-cupping ultrasound image from the condition of (c).

An ultrasound device (ProSound A7, Hitachi Healthcare Americas, Twinsburg, OH) with the ultrasound probe of the frequency of 17–21 MHz (UST-5412; Hitachi Health care Americas) was used to measure the B-mode ultrasound images of the triceps muscles. The 17 MHz ultrasound signal was selected for this study due to the reason that the limb muscle may be thicker than 6 cm; thus, the lowest available ultrasound frequency was selected. During experiments of all participants, the ultrasound gain was kept the same to avoid the influence of subcutaneous fat tissue thickness. Although it is well known that muscle echo is largely affected by gain and texture analysis is not significantly affected, we still kept the setting across all measurements. The same operator collected all ultrasound images. All recorded ultrasound images were saved in the format of the Digital Imaging and Communications in Medicine (DICOM). Examples of gray-scale ultrasound images are provided in Fig 1.

**Fig 2:**
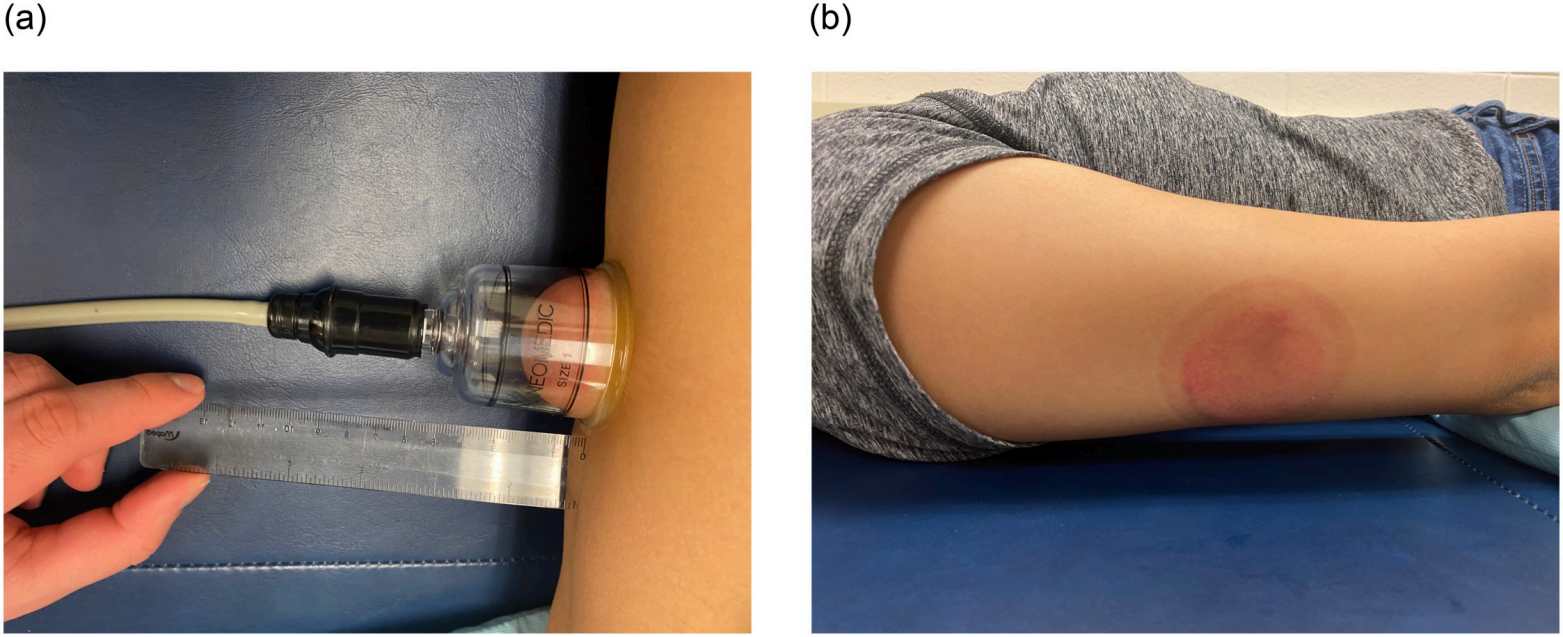
Experimental setup during cupping therapy. (a) The soft tissue over the triceps area is sucked into the cup during cupping therapy using an electronic cupping device. (b) The redness over the cupping area appears after cupping therapy.

To create negative pressure inside the cupping cup, an electrical suction machine (P1000-PCS, California Medical Device Manufacturing Facility, CA) was used to achieve the pre-determined magnitude of negative pressure [31]. The advantage of using an electrical suction device is that we could apply the same magnitude of negative pressure across all research participants. This suction method does not incorporate the skin piercings or blood-letting therefore it is a safer treatment procedure (also called dry cupping). The cup size was selected at the inner diameter of 45 mm and the outer diameter of 53 mm to allow a large cup size for this study [7, 32, 33] (Fig 2).

### Cupping protocols

Currently, there is no standardized guideline for negative pressure and duration to be applied in cupping therapy for specific musculoskeletal impairment. Therefore, we decided to choose −225 and −300 mmHg as the magnitudes of negative pressure of cupping therapy for reducing muscle stiffness from the previous studies [8, 34]. The cupping duration was selected between 5 to 10 min according to several clinical studies [8, 35]. The selections of these negative pressures and durations are based on common settings of cupping therapy for effectively inducing muscular and microvascular responses [8, 9, 29]. Therefore, these settings were selected to ensure the intensity of cupping therapy would be sufficient to induce changes in muscle texture [8].

We chose the triceps muscle as the treatment area to investigate the effect of cupping therapy for muscle stiffness relief. Xiaoluo acupoint which is located at the triceps is usually used as a common treatment point for alleviating pain around the upper limb according to traditional Chinese medicine practice without evidence [8]. The location of Xiaoluo was determined from about the lower 40% from the olecranon to acromion; and a light pressure was applied on the area of the triceps to confirm the location. In order to cover the Xiaoluo area, the cup size at 45-mm in inner diameter was used to ensure that the acupoint was covered inside the cupping cup [7, 8]. In addition, our research group has recently corroborated the finding that cupping at Xiaoluo can accelerate skin blood flow and reduce muscle stiffness [7, 8].

### Procedures

The experiments were conducted in the Rehabilitation Engineering Laboratory at the University of Illinois at Urbana-Champaign. Each participant received four cupping protocols at four different days to minimize the carryover effort. Before the experiment, each participant had to accustom to the room temperature for 30 minutes. The participant was in a supine position with his/her elbow in full extension, the forearm in full pronation, and the wrist in the neutral position (Fig 2). Five ultrasound images over the triceps belly were recorded to establish the baseline condition of the triceps (pre-cupping). Then, cupping therapy was applied to the center of Xiaoluo acupoint of the triceps muscle area of the dominant arm. The order of allocation of 4 cupping protocols was according to the pre-determined order. After that, the cup was removed and the ultrasound image measurements were taken another 5 times (post-cupping). There were a total of 480 ultrasound images collected for this study based on 12 subjects, 10 measurements per protocol (5 pre-cupping and 5 post-cupping images) for 4 protocols.

### Data analysis

Musculoskeletal ultrasound is a popular assessment tool in sports and rehabilitation for assessing muscle structure, such as thickness, cross-sectional area and fascicle pennation angle. Muscle texture analysis is an emergent tool for quantifying spatial relative features within the muscle. Molinari et al. demonstrated that the second-order (eg. texture analysis) is more sensitive than the first order [36]. The B-mode ultrasound images were used to determine the accurate location of humerus of all images. The pixel location of humerus on the B-mode images was used to recognize the ROI. ROI in this study was defined as a rectangular area through the skin surface to the humerus in each image. The width of ROI was defined as the central 90% of the ultrasound image to eliminate the distortion margin. The depth was defined as the range from the skin to the edge of humerus. The ROI of each image was computed using texture analysis. The texture analysis of B-mode ultrasound images was implemented using GLCM, also known as the gray-level spatial dependence matrix [37]. GLCM quantifies the texture of an ultrasound image by using the ultrasound image to create a GLCM and then extracting statistical measures. In this study, four parameters were calculated including the contrast, correlation, energy and homogeneity. Although there are more than 10 texture features, we selected four features in this study to cover the major types of texture features. Although these texture features are highly correlated, it is usually recommended to select several texture features to characterize muscle compositions and quality because the exact physiological meanings of these texture features have not been established [23, 25].

Contrast texture measures the local variations in the GLCM. Contrast parameter is the result of gray level dispersion on an image. According to Wilkinson’ study [38], a lower value of contrast texture indicates regions have high homogeneity (Eq 1).


$$Contrast=\overset{n-1}{\underset{i,j=0}{\sum }}P_{i,j}{\left(i-j\right)}^{2}$$ (1)

Correlation texture refers to the joint probability occurrence of the pixel pairs. A greater value of correlation index indicates regions have similar gray levels, as a high homogeneity (Eq 2).


$$Correlation=\overset{n-1}{\underset{i,j=0}{\sum }}P_{i,j}\left[\frac{\left(i-\mu_{i}\right)\times \left(j-\mu_{j}\right)}{\sqrt{\sigma_{i}^{2}\sigma_{j}^{2}}}\right]$$ (2)

Energy texture, also known as uniformity or the angular second moment, provides the sum of squared elements in the GLCM. When an ultrasound image is homogeneous, the value of energy index is higher (Eq 3).


$$Energy=\overset{n-1}{\underset{i,j=0}{\sum }}{\left(P_{i,j}\right)}^{2}$$ (3)

Homogeneity texture, also known as the inverse difference moment, measures the closeness of the distribution of elements in the GLCM. When an ultrasound image is homogeneous, the value of homogeneity index is higher (Eq 4).


$$Homogeneity=\overset{n-1}{\underset{i,j=0}{\sum }}\frac{P_{i,j}}{1+{\left(i-j\right)}^{2}}$$ (4)

The texture index values were calculated by dividing the post-cupping value by the pre-cupping value, resulting in normalized texture values. In this study, we did not assess the reliability of texture analysis on assessing muscle composition and quality and cited previous studies to support that texture analysis is a reliable method [24, 25]. The calculation was carried out by Matlab and the Image Processing Toolbox (2019R, MathWorks, Inc., Natick, MA). The effects of the pressure and duration factors and the interaction effect between above two factors were analysis by two-way repeated measures analysis of variance (ANOVA). The test of sphericity was used to examine whether the assumption of a normal distribution was met. Also, the pre-cupping images of four protocols were examined to determine whether a significant difference exits. If the main effect exists, the paired-t test with the Bonferroni correction was used to compare the difference between two conditions for the post-hoc comparisons [39]. The *P* values below 0.05 was set as the significance level, and all statistical tests were performed by SPSS (Version 29, IBM).

## Results

In this study, a total of 12 participants from the students and the staff of the University of Illinois were included for the final analysis. The demographic data were: 5 males and 7 females, 8 Asians and Asian Americans and 4 Caucasians, age 25.42 ± 4.9 years old, body height 1.7 ± 0.1 m, body weight 75.1 ± 18.7kg, body mass index 25.2 ± 4.4 kg/m^2^, arm circumference 28.9 ± 3.6 cm, systolic blood pressure 112.8 ± 13.7 mmHg, diastolic blood pressure 69.3 ± 8.6 mmHg, and heart rate 74.4 ± 7.8 beats/min. All participants did not report any adverse events from participating in this study.

**Fig 3:**
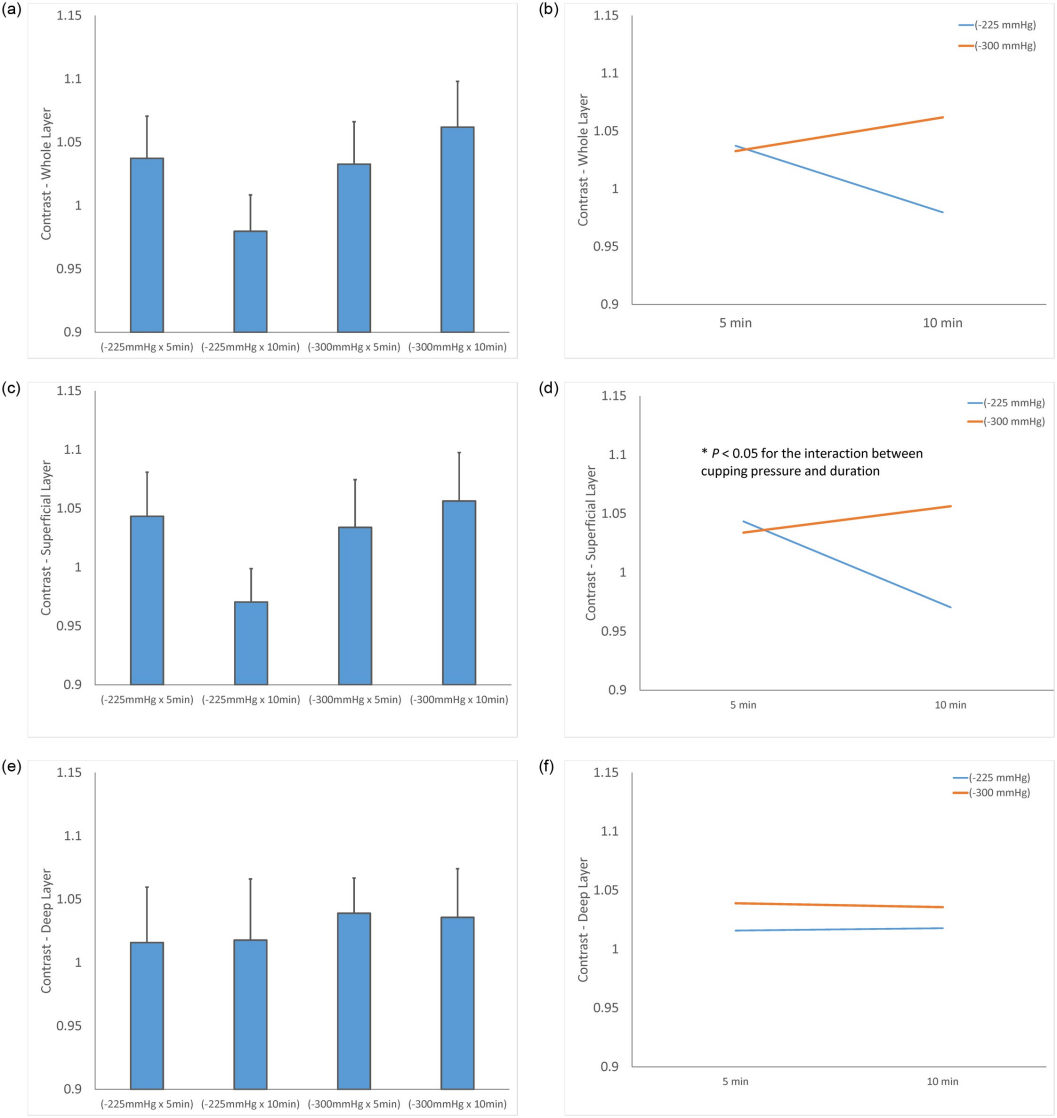
Normalized contrast texture of the triceps muscle (post-cupping / pre-cupping contrast). (a) Contrast values of the whole layer. (b) Interaction effect between cupping pressure and duration on contrast values of the whole layer. (c) Contrast values of the superficial layer (d) Interaction effect between cupping pressure and duration on contrast values of the superficial layer. (e) Contrast values of the deep layer. (f) Interaction effect between cupping pressure and duration on contrast values of the deep layer. (* *P* < 0.05).

**Fig 4:**
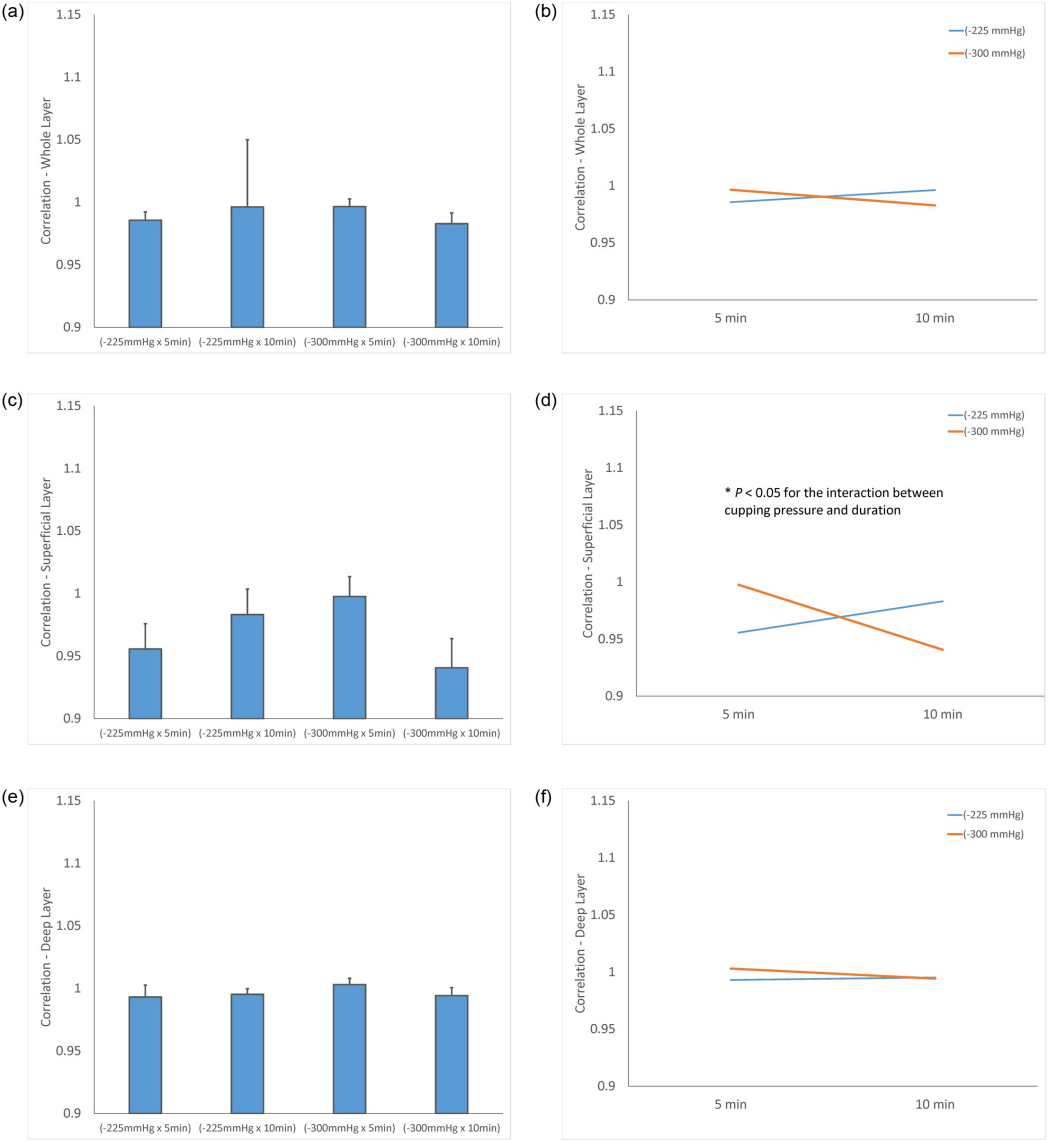
Normalized correlation texture of the triceps muscle (post-cupping / pre-cupping correlation). (a) Correlation values of the whole layer. (b) Interaction effect between cupping pressure and duration on correlation values of the whole layer. (c) Correlation values of the superficial layer. (d) Interaction effect between cupping pressure and duration on correlation values of the superficial layer. (e) Correlation values of the deep layer. (f) Interaction effect between cupping pressure and duration on correlation values of the deep layer. (* *P* < 0.05).

The test of sphericity indicates that all contrast, correlation, energy and homogeneity textures before and after cupping therapy do not violate the normal distribution assumption. Therefore, parametric statistics were used. No difference was observed between any comparisons among texture of pre-cupping ultrasound images of 4 protocols. Two-way repeated measures ANOVA found a significance in the contrast and correlation textures but not energy and homogeneity textures (Table 1). The detailed results are reported for contrast and correlation textures in Figs 3 and 4.

**Table 1:** Statistical results of the two-way ANOVA with repeated measures on the interaction and main effects of the pressure and duration factors of cupping therapy.

Factor	Texture Variable	Layer	F Value	*P* Value	Effect Size
**Pressure × Duration**	**Contrast**	Whole Layer	3.911	0.074	0.262
		Superficial Layer	5.434	0.040*	0.331
		Deep Layer	0.010	0.924	0.001
	**Correlation**	Whole Layer	3.561	0.086	0.245
		Superficial Layer	6.274	0.029*	0.363
		Deep Layer	0.565	0.468	0.049
	**Energy**	Whole Layer	2.554	0.138	0.188
		Superficial Layer	2.217	0.165	0.168
		Deep Layer	0.147	0.708	0.013
	**Homogeneity**	Whole Layer	0.074	0.791	0.007
		Superficial Layer	0.263	0.618	0.023
		Deep Layer	0.069	0.798	0.006
**Pressure**	**Contrast**	Whole Layer	0.896	0.364	0.075
		Superficial Layer	0.804	0.389	0.068
		Deep Layer	0.127	0.729	0.011
	**Correlation**	Whole Layer	0.033	0.858	0.003
		Superficial Layer	0.023	0.881	0.002
		Deep Layer	0.835	0.381	0.071
	**Energy**	Whole Layer	0.401	0.540	0.035
		Superficial Layer	0.080	0.783	0.007
		Deep Layer	0.878	0.369	0.074
	**Homogeneity**	Whole Layer	0.273	0.612	0.024
		Superficial Layer	0.024	0.878	0.002
		Deep Layer	0.233	0.639	0.021
**Duration**	**Contrast**	Whole Layer	0.297	0.597	0.026
		Superficial Layer	0.738	0.409	0.063
		Deep Layer	0.000	0.983	0.000
	**Correlation**	Whole Layer	0.063	0.807	0.006
		Superficial Layer	0.558	0.471	0.048
		Deep Layer	0.334	0.575	0.029
	**Energy**	Whole Layer	0.035	0.856	0.003
		Superficial Layer	0.661	0.433	0.057
		Deep Layer	0.246	0.630	0.022
	**Homogeneity**	Whole Layer	0.188	0.673	0.017
		Superficial Layer	1.177	0.301	0.097
		Deep Layer	0.122	0.733	0.011

### Contrast texture

In contrast texture, the overall layer of the triceps muscle shows an increase in the three protocols (−225 mmHg for 5 min, −300 mmHg for 5 min, and −300 mmHg for 10 min), but not in the protocol of −225 mmHg for 10 min in Fig 3. The two-way repeated measures ANOVA indicates that there are no interactions between the pressure and duration factors on normalized contrast texture of the overall layer (F = 3.911, *P* = 0.074) (Table 1). The normalized muscle texture of triceps muscle is 1.037 ± 0.115 (−225 mmHg for 5 min), 1.033 ± 0.116 (−300 mmHg for 5 min), and 1.062 ± 0.126 (−300 mmHg for 10 min) of pre-cupping texture, and is 0.980 ± 0.100 (−225 mmHg for 10 min) of pre-cupping texture (Fig 3A). Under −225 mmHg, normalized contrast texture of the triceps after 5-min cupping (1.037 ± 0.115) is higher than 10-min cupping (0.980 ± 0.100, *P* = 0.051) (Fig 3B, Table 1).

In contrast texture, the superficial layer of the triceps muscle shows a significant increase in the three protocols (−225 mmHg for 5 min, −300 mmHg for 5 min, and −300 mmHg for 10 min), but not in the protocol of −225 mmHg for 10 min in Fig 3. The two-way repeated measures ANOVA indicates that there is an interaction between the pressure and duration factors on normalized contrast texture of the superficial layer (F = 5.434, *P* = 0.040) (Table 1). The normalized muscle texture of triceps muscle is 1.0434 ± 0.130 (−225 mmHg for 5 min), 1.0339 ±0.1407 (−300 mmHg for 5 min), and 1.0563 ±0.1432 (−300 mmHg for 10 min) of pre-cupping texture, and is 0.9704 ±0.0985 (−225 mmHg for 10 min) of pre-cupping texture (Fig 3C). Under −225 mmHg, normalized contrast texture of the triceps after 5-min cupping (1.0434 ± 0.130) is higher than 10-min cupping (0.9704 ±0.0985, *P* = 0.051) (Fig 3D, Table 1).

In contrast texture, the deep layer of the triceps muscle shows an increase in the four protocols (−225 mmHg for 5 min, −225 mmHg for 10 min, −300 mmHg for 5 min, and −300 mmHg for 10 min) in Fig 3. The two-way repeated measures ANOVA indicates that there are no interactions between the pressure and duration factors on normalized contrast texture of the deep layer (F = 0.010, *P* = 0.924) (Table 1). The normalized muscle texture of triceps muscle is 1.0158 ± 0.1519 (−225 mmHg for 5 min), 1.0178 ± 0.1672 (−225 mmHg for 10 min), 1.039 ± 0.0964 (−300 mmHg for 5 min), and 1.0357 ± 0.1332 (−300 mmHg for 10 min) of pre-cupping texture (Fig 3E). Under −225 mmHg, normalized contrast texture of the triceps after 5-min cupping (1.0158 ± 0.1519) is lower than 10-min cupping (1.0178 ± 0.1672, *P* = 0.051) (Fig 3F; Table 1).

### Correlation texture

In correlation texture, the overall layer of the triceps shows a decrease in the four protocols (−225 mmHg for 5 min, −225 mmHg for 10 min, −300 mmHg for 5 min, and −300 mmHg for 10 min) in Fig 4. The two-way repeated measures ANOVA indicates that there are no interactions between the pressure and duration factors on normalized correlation texture of the overall layer (F = 3.561, *P* = 0.086) (Table 1). The normalized correlation texture of triceps muscle is 0.9856 ± 0.0229 (−225 mmHg for 5 min), 0.9962 ± 0.1863 (−225 mmHg for 10 min). 0.9965 ± 0.0212 (−300 mmHg for 5 min), and 0.9828 ± 0.0299 (−300 mmHg for 10 min) of pre-cupping texture (Fig 4A). Under −225 mmHg, normalized correlation texture of the triceps after 5-min cupping (0.9856 ± 0.0229) is lower than 10-min cupping (0.9962 ± 0.1863, *P* = 0.051) (Fig 4B, Table 1).

In correlation texture, the superficial layer of the triceps shows a significant decrease in the four protocols (−225 mmHg for 5 min, −225 mmHg for 10 min, −300 mmHg for 5 min, and −300 mmHg for 10 min) in Fig 4. The two-way repeated measures ANOVA indicates that there is an interaction between the pressure and duration factors on normalized correlation texture of the superficial layer (F = 6.274, *P* = 0.029) (Table 1). The normalized correlation texture of triceps muscle is 0.9556 ± 0.07 (−225 mmHg for 5 min), 0.9831 ± 0.0708 (−225 mmHg for 10 min). 0.9976 ± 0.055 (−300 mmHg for 5 min), and 0.9406 ± 0.0809 (−300 mmHg for 10 min) of pre-cupping texture (Fig 4C). Under −225 mmHg, normalized correlation texture of the triceps after 5-min cupping (0.9556 ± 0.07) is lower than 10-min cupping (0.9831 ± 0.0708, *P* = 0.051) (Fig 4D, Table 1).

In correlation texture, the deep layer of the triceps shows a decrease in the three protocols (−225 mmHg for 5 min, −225 mmHg for 10 min, and −300 mmHg for 10 min), but not in the protocol of −300 mmHg for 5 min in Fig 4. The two-way repeated measures ANOVA indicates that there are no interactions between the pressure and duration factors on normalized correlation texture of the deep layer (F = 0.565, *P* = 0.468) (Table 1). The normalized correlation texture of triceps muscle is 0.993 ± 0.0333 (−225 mmHg for 5 min), 0.9952 ± 0.01579 (−225 mmHg for 10 min), 1.003 ± 0.01732 (−300 mmHg for 5 min), and 0.9942 ± 0.02224 (−300 mmHg for 10 min) of pre-cupping texture (Fig 4E). Under −225 mmHg, normalized correlation texture of the triceps after 5-min cupping (0.993 ± 0.0333) is lower than 10-min cupping (0.9952 ± 0.01579, *P* = 0.051) (Fig 4F; Table 1).

## Discussion

Our study provides the first attempt by using gray-scale ultrasound with texture analysis to investigate the efficacy of different negative pressures and durations of cupping therapy on muscle quality. The novelty of this study is that both the contrast and correlation texture features were sensitive to quantify the changes of muscle quality of the triceps muscle after cupping therapy. The results demonstrated that the interaction between the pressure and duration factors of cupping therapy significantly increased the contrast texture and significantly decreased the correlation texture of the superficial layer of the triceps muscle after cupping therapy. Our finding support the use of texture analysis of B-mode ultrasound images for assessing the changes in muscle texture after cupping therapy.

In this study, we demonstrated that contrast and correlation features are sensitive to detect changes in muscle quality after cupping therapy but not energy and homogeneity features. The finding has potential to improve clinical practice on assessing muscle quality as well as to develop a method for assessing underlying mechanisms of action of cupping therapy. Clinically, muscle quality is usually assessed by gray-scale ultrasound. For biomedical image analysis, there are two categories of analysis for these images including intensity analysis and texture analysis. Image intensity relates to the statistical distribution of the pixel values inside a defined ROI. Traditional quantitative measures include mean, variance, skewness, and kurtosis. Although these measures are useful to characterize the image content, these measures cannot quantify the spatial relationships between pixel values of a gray-scale ultrasound image. This may partly explain why ultrasound imaging is not a widely used imaging tool for assessing musculoskeletal interventions [38]. Due to the limitation of muscle echogenicity, texture analysis has been recently introduced to assess muscle quality from ultrasound images [25, 26, 36, 38, 40]. Wilkinson et al. used five features of texture analysis including energy, entropy, homogeneity, correlation and contrast to assess muscle quality changes in patients with chronic kidney disease [38]. The authors found that muscle function is associated with greater ultrasound texture values (greater correlation and lower contrast).

In this study, contrast texture increased in the 3 protocols in both superficial and overall layers, except −225 mmHg for 10 min; and correlation texture decreased in the superficial layer of the triceps in 4 protocols. The finding represents that the muscle texture altered by cupping therapy can be quantified by texture analysis, which may reflect potential mechanisms of action of cupping therapy to manage musculoskeletal impairment. The altered property induced by cupping therapy may affect mechanical environment surrounding painful and injury areas for reducing pain and promoting repair process. This speculation is not directly supported by this study. However, the use of texture analysis of muscle ultrasound images provide evidence that cupping therapy can effectively change these texture features, such as contrast and correlation texture. In the previous studies [7, 29], cupping therapy has been demonstrated to reduce stiffness in the deep layer of the soft tissue. However, our finding indicates that cupping therapy is effective on increasing contrast texture and reducing correlation texture in the superficial layer of the soft tissue. These findings may appear conflicting with each other. However, these results are actually reasonable based on the tissue properties in response to mechanical stress, such as negative pressure-based cupping therapy. The deep layer of the soft tissue (eg. biceps and triceps areas) mainly consists of the muscle that has been shown to reduce stiffness under prolonged stress but not het superficial layer [7]. However, the superficial layer of the soft tissue consists of the skin, subcutaneous tissue, fat and some muscles that may be seen as a complex material. When under cupping therapy, different compositions (eg. fat and muscles) may exhibit very different responses, thereby texture features of the superficial layer may show a significant change but not the deep layer. Further research is needed to confirm our findings to see whether cupping therapy is effective on reducing stiffness of the deep layer and is effective on changing texture of the superficial layer of the soft tissue. This could also imply that people with different body fat may exhibit various responses to cupping therapy. To sum up, a comprehensive understanding of the effect of body mass index and body shape on the soft tissue response to cupping therapy is needed to improve efficacy and effectiveness of cupping therapy.

Four cupping therapy protocols were examined in this study. Overall, all four protocols appear effective on changing texture of the soft tissue except −225 mmHg for 10 min on contrast texture. This could indicate that −300 mmHg is more effective than −225 mmHg because both protocols consisting of −300 mmHg reached a significant change. Under −225 mmHg, our results indicate that 5 min is more effective than 10 min on changing contrast texture. This is actually consistent with previous studies indicating that a shorter duration of cupping therapy (eg. 5 min is better than 10 min) on improving skin blood flow [8]. It has been reported that junior clinicians who apply cupping therapy using the fire approach may induce a smaller negative pressure, such as −225 mmHg. If this is the case, our results suggest these junior clinicians to apply cupping therapy at a shorter duration for a more effective change in texture of the treated soft tissue.

Our results demonstrated that cupping therapy was effective on increasing contrast texture and decreasing correlation texture of the superficial layer of the triceps. This actually does not meet our hypothesis that cupping therapy could reduce “contrast” and increase “correlation” among soft tissues of the triceps muscle for improving homogeneous status. Image homogeneity likely indicates lower muscle inflation of fat and fibrosis; and following this principle, an effective intervention could increase homogeneous among tissues to avoid stress concentration in certain areas to reduce risk for musculoskeletal injury [24, 27]. Because we consider cupping therapy as a beneficial intervention, an increased contrast observed in this study counters this hypothesis [24, 27]. Although cupping therapy has been demonstrated to improve microcirculation for accelerating muscle recovery, it is unclear whether cupping therapy can improve texture characteristics among treated soft tissues. In this study, the defined superficial layer consists of the skin, fat and muscles and the defined deep layer mainly consists of the muscle. The negative pressure of cupping therapy could effectively stretch these soft tissues in the superficial layer of the triceps muscle for changing texture characteristics. Tham et al. demonstrated that the stress is larger at the area close to the skin using a finite element model [11]. This could explain that the superficial layer was significantly affected and not the deep layer after cupping therapy. Nevertheless, our finding raise a question to examine whether cupping therapy could cause a minor damage to soft tissues, therefore, reducing homogeneity for a higher contrast and a lower correlation of the superficial layer of the triceps.

A unique strength of using texture analysis is supported by the finding that the human visual system possesses neurons that are selectively sensitive to directional spatial frequency [41]. As shown in Fig 2, these texture changes caused by cupping therapy may not be easily detected by visual examination of clinicians; and the use of texture analysis could reveal and quantify these changes caused by cupping therapy. The most common method for texture analysis is using GLCM to quantify the relationship between neighboring pixel’s intensities and is based on the use of second-order statistics of the grayscale image histograms. If an image texture increase (eg. the local pixel intensity variations increase), the off-diagonal values in the GLCM become larger. The use of texture analysis can sum up changes in these ROI. Thus, it is important to define the area treated by cupping therapy with an appropriate placement of ultrasound measurement. Computer-based quantitative methods such as texture analysis has a potential to reduce image interpretation errors, especially in the first-order statistics (eg. ultrasound echo intensity analysis).

There are several limitations in this study. First, the research participants were from a homogenous group, which were healthy with normal body mass index. Thus, the findings of this study may not be generalized to people who are with higher or lower body mass index because of the potential influence of fat tissue in response to cupping therapy. Moreover, there were more females than males that may influence the results. It is unknown whether the gender effect would affect the efficacy of cupping therapy on muscle quality. Third, the intervention protocol of cupping therapy tested in this study included 4 common combinations of pressure (-225 and -300 mmHg) and duration (5 and 10 min). Different combinations of pressure and duration of cupping therapy need to be evaluated to validate our findings. Fourth, we did not conduct a pilot study to determine the test-retest reliability of texture analysis of B-mode ultrasound images and only cited the literature to indicate that this method is a reliable method. Future studies may need to establish the reliability of texture analysis to assess muscle composition and quality. Last, the increased contrast and the decreased correlation of the triceps after cupping therapy may imply that cupping therapy may cause damage to the soft tissues. Future studies may need to examine the exact meaning of these changed texture of the muscle after cupping therapy.

## Conclusion

Our results demonstrate that contrast and correlation features of texture analysis of gray-scale ultrasound images have higher discrimination accuracy to quantify the effect of cupping therapy on muscle quality compared to energy and homogeneity features. In addition, texture analysis revealed that the superficial layer of the triceps is significantly under influence by the interaction between cupping pressure and cupping duration. The protocols tested in this study demonstrated that cupping therapy at −300 mmHg at both 5 and 10 min is effective on changing texture of the treated soft tissue. The results using texture analysis of gray-scale ultrasound images indicates that cupping therapy could effectively change muscle quality of the superficial layer of the triceps. Physiological meanings of these changes in texture require further investigation.
